# Supplementary material for: Evidence-based economic analysis demonstrates that ecosystem service benefits of water hyacinth management greatly exceed research and control costs
Source: PeerJ. 2018 May 23;6:e4824. doi: 10.7717/peerj.4824 (PMC5970557; doi:10.7717/peerj.4824)
Supplement: Supplemental Information 1 [file peerj-06-4824-s001.pdf]

Appendix A: Development of the spatio-temporal spread model

Dong Liang

University of Maryland Center for Environmental Science, Chesapeake Biological Lab, Solomons, MD

dliang@umces.edu

Associated article: Comprehensive data and parsimonious models enable evidence-based economic analysis of ecosystem service benefits of invasive species management options*. L. Wainger, N. Harms, C. Magen, D. Liang, G. Nesslage, A. McMurray, and A. Cofrancesco.*

Introduction

Modeling spread of invasive species involves estimating density-dependent growth through time and spatial dispersion (Robinet et al. 2012). Stochastic spread models have been used to model growth of invasive species (e.g., Hooten et al. 2008), but are analytically complex and may not provide generalizable results. Robinet et al. (2012) developed a set of parsimonious models that linked logistic population growth with environmental niche maps. The approach is analytically tractable and flexible to accommodate a large variety of pest risk assessments, as might be conducted for management decision analysis. Here, we adapted one of the Robinet et al. models (Model D) to estimate the population dynamics of water hyacinth with biological control and to estimate the cost and benefit of biological control. In the following sections, we describe the study area, the spatio-temporal spread model, adaption of the model to the water hyacinth data, and the statistical calibration procedure.

Study Area

The study area consists of a raster with 1,914 grid cells (i=1,…,1914) within water bodies for the state of Louisiana. These cells were generated by rasterizing the 2006 TIGER census water body polygons (U.S. Census Bureau 2006) and represented the spatial basis for the spread model.

Seventy-six cells were assigned the values of historical reports of water hyacinth occurrence from 1939 to 2000 (Figure A-1, Pam Fuller, USGS, 2001, pers. comm.). The grid cells were parameterized by CLIMEX (Sutherst et al. 2007) niche maps of climate suitability and host presence. The global CLIMEX outputs representing the climate suitability (E) and growth potential (G) were downscaled and rasterized into 1 × 1 km cells using ordinary Kriging. Then each cell i in the state of Louisiana water body raster was assigned the value of the closest corresponding CLIMEX suitability E_i and growth G_i indices.

Spatio-Temporal Spread Model Structure

The spatio-temporal spread model encompassed three submodels, a) water hyacinth habitat suitability by grid cell; b) biomass growth per cell; and c) spread across cells per time period. We used a parsimonious model (adapted from Eqns. (2-3, 4-6) Robinet et al. 2012) to create these three models. Water hyacinth habitat suitability was designated by CLIMEX niche maps (Kriticos, pers comm) that characterized likelihood of occurrence and maximum density of water hyacinth per cell. Growth per cell per year was characterized by a logistic population growth model (Wilson et al. 2005). The growth

model was parameterized by cell specific carrying capacity (K_i) and intrinsic growth rate (λ_i). The CLIMEX growth index *Gi* was used to model the spatial variation in intrinsic growth rates among cells such that $\lambda_i = \lambda \times G_i$ (see Eqs. (2-3) Robinet et al. 2012). The CLIMEX climate suitability *Ei* index determined the cell specific carrying capacity K_i (Figure A-2), which was computed based on the statewide maximum observed extent of 6,177 thousand acres (Manning 1979). Specifically $K_i = K \times E_i$ such that $K \times \sum E_i$ = 6,177. The *K* value was assumed to be known and fixed across the years.

After applying logistic growth per grid cell, the spatial spread across grid cells was modeled through a two dimensional *t* kernel; with scaling parameter *u* determining the width of kernel, and a degree of freedom parameter *p* modelling the chance of a long distance spread (Eqns. 4-6, Robinet et al. 2012). Given the statewide initial occurrences of the water hyacinth, we used an isotropic (non-directional) kernel. However, the full kernel matrix was a dense 1,904 × 1,904 matrix and therefore computationally challenging to manipulate in the simulation. Therefore, we only considered spread to the fifty nearest neighbors to approximate the spread in each growing season, which was deemed sufficient to approximate the full kernel matrix. The resulting kernel matrix was sparse, and can be multiplied at O(*nlogn*) time complexity (the amount of time the algorithm takes to run as a function of the input size *n*) using sparse matrix method to facilitate computationally efficient model simulation.

We initialized the population by distributing 1,000 acres of water hyacinth across the study area by assigning a density of 0.28% to each cell where there was an initial reported occurrence. The intrinsic growth rate of the logistic growth model was then estimated from spring and fall cover data from 1975 to 2013 (Louisiana Department of Wildlife and Fisheries, pers comm). Finally, spread across cells was simulated using a kernel density function calibrated using spatial extent data and a statistical optimization routine. The calibrated model was used to simulate the spread of water hyacinth under biocontrol and no-control scenarios.

Model Adaption to Louisiana Water Hyacinth Data

In addition to changes described above, the spatio-temporal spread model was further adapted to Louisiana data sources in three primary ways. First, the model was used to estimate the intrinsic growth rate and account for over-winter mortality from multiple data sources. Second, the model was initiated using the locations and timing of historic occurrences of water hyacinth as compiled from many sources (P. Fuller, USGS, pers comm). The data represent documented treatment sites in Louisiana from 1974-2013 and may not have been complete. Third, the lag between the initial occurrences and the observations reported in Spring 1975 was included in the sub-models as a tuning parameter. According to historical reports, the range for this lag between establishment and observation was thought to be between 40 and 70 years. The intrinsic growth rate during this initial growth period was assumed to be stationary and similar to those between 1975 and 1978 (Manning 1979).

The water hyacinth annual occurrence reports include spring and fall acreages per year since 1974. The difference between fall and spring acreages was used to estimate overwinter natural morality. We assumed that none of the winter mortality was due to the change in the growth rate and the mortality parameter was estimated independently from the spread model as the ratio between the observed spring acreage and the predicted acreage for the previous fall. Thus, overwinter mortalities served as tuning parameters to reset the model simulations every year to the observed spring acreages, so only annual growth between spring and fall acreages were used in estimating the logistic growth and spread model.

For the logistic population growth model, we allowed the annual intrinsic growth rates to vary in time to provide a better fit to the observed data (Nesslage et al. 2016). For the biocontrol scenario, the annual intrinsic growth rate was assumed constant prior to 1975 (Manning 1979), and then was allowed to vary from year to year until 2013 to reflect effects of control.

Model Calibration

The spatio-temporal spread model was calibrated using the maximum likelihood estimation method. We assumed a log-normal distribution for the observed statewide fall acreage between 1975 and 2013. The model was initiated with the map of historic occurrences and then run to generate predicted state-wide fall acreage estimates between 1975 and 2013. The predicted statewide fall acreages were then compared with the reported fall acreages by sum of squared difference (SSE) on a natural log scale. Numerical optimization was then used to minimize the SSE between model prediction and the actual acreage by varying logistic population growth and spatial dispersal parameters. The calibration predicts the observed fall acreages with R^2=0.78, indicating high quality in the calibration (Figure A-3).

Optimizations were repeated with different choices of the tuning parameters and a lag period of 40 years was found to best match (minimized SSE) the data gap between the initial occurrences and the recorded acreages after fall 1975. To avoid sensitivity to the initial values, the optimization process was restarted from randomly chosen initial values. At convergences, the set of parameters that gave a minimal SSE was reported as the final estimate. The *nlminb* method implemented in the R *optimx* package (Nash & Varadhan 2011) was used for the optimization. Convergence was checked using the built-in criteria.

Model Simulation Results

With Treatment Scenario: This simulation represents the scenario with active statewide biocontrol and human intervention, which began after 1979. Figure A-4 shows the time-varying growth parameters estimated from 1975 – 2013 (representing effects of control on growth rate) and the static growth rate for 1934 - 1974. These estimated parameter values were used to generate the cell-specific acreages at each spring and fall between 1934 and 2013 over the state of Louisiana (Figure A-5).

No Treatment Scenario: Assuming that the intrinsic growth rates prior to 1978 represent the scenario without statewide bio-control (Manning 1979), the estimated intrinsic growth rates during this period were re-sampled with replacement to fill in the period between 1979 and 2013. The annual overwinter mortality parameters between 1979 and 2013 were assigned the same empirical values as the with treatment scenario. The carrying capacity, spatial spread kernel, and lag tuning parameters were assumed to be the same as those used in the with treatment scenario. These estimated parameter values were used to generate the cell-specific acreages for each spring and fall between 1934 and 2013.

Control Effects: The effects of control do not start to emerge until 1978 when the without control scenario shows a dramatic increase in water hyacinth coverage but the control scenario shows a continuing decline and then stabilization of cover at a low level (Figure A-5). In contrast, the without control scenario, shows a sharp increase in coverage that varies annually but remains close to the carrying capacity from 1990 onward. Both scenarios show decreases 1975-1980 due to the severe over-winter mortality (Nesslage et al. 2016). The annual variability in the without control results from occasional over-winter mortality.

The maps of model outputs of the no-control and control scenarios also illustrate striking differences between scenarios in the south of the state (Figure 2). The differences between northern and southern regions of the state can be attributed to differences in habitat suitability, resulting in a lower growth rate and lower carrying capacity in the north of the state compared to the south which has the warmer water temperatures preferred by water hyacinth.

References

Hooten, M.B. and Wikle, C.K., 2008. A hierarchical Bayesian non-linear spatio-temporal model for the spread of invasive species with application to the Eurasian Collared-Dove. *Environmental and Ecological Statistics*, *15*(1), pp.59-70.

Manning, J.H., 1979. Establishment of water hyacinth weevil populations in Louisiana. *Journal of Aquatic Plant Management*, *17*, pp.39-41.

Nash, J.C., R. Varadhan (2011). Unifying Optimization Algorithms to Aid Software System Users: optimx for R. *Journal of Statistical Software*, 43(9), 1-14. URL http://www.jstatsoft.org/v43/i09/.

Nesslage, G., L. Wainger, N. Harms, A. Cofrancesco. 2016. Quantifying the population response of invasive water hyacinth, Eichhornia crassipes, to biological control and winter weather in Louisiana, USA. *Biological Invasions*, 18(7): 2107-2115.

Robinet, C., Kehlenbeck, H., Kriticos, D.J., Baker, R.H., Battisti, A., Brunel, S., Dupin, M., Eyre, D., Faccoli, M., Ilieva, Z. and Kenis, M., 2012. A suite of models to support the quantitative assessment of spread in pest risk analysis. *PLoS One*, *7*(10), p.e43366.

Wilson, J.R., Holst, N. and Rees, M., 2005. Determinants and patterns of population growth in water hyacinth. *Aquatic Botany*, *81*(1), pp.51-67.

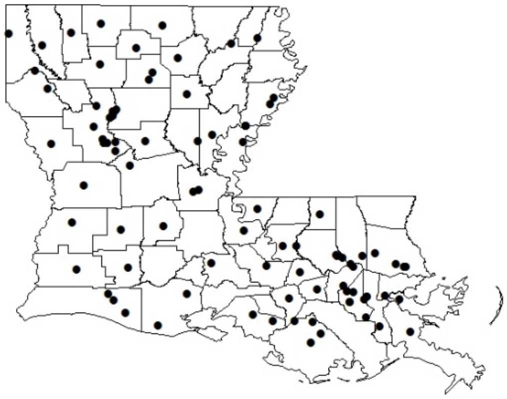

Figure A-1: Map of historic water hyacinth occurrences

Source: Pam Fuller, USGS, 2014, pers. comm.

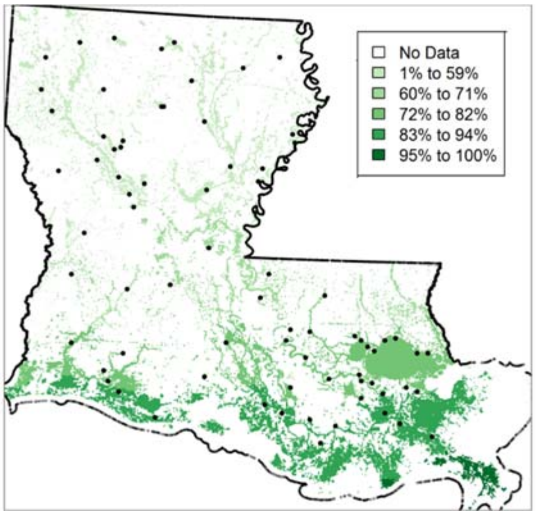

Figure A-2: Water hyacinth carrying capacity, as derived from CLIMEX outputs

Note: Water bodies in the northern part of the state can only be partially covered by water hyacinth due to low habitat suitability. The black dots represent the 76 occurrences derived from data in Figure A-1 that were used to initiate the spread model

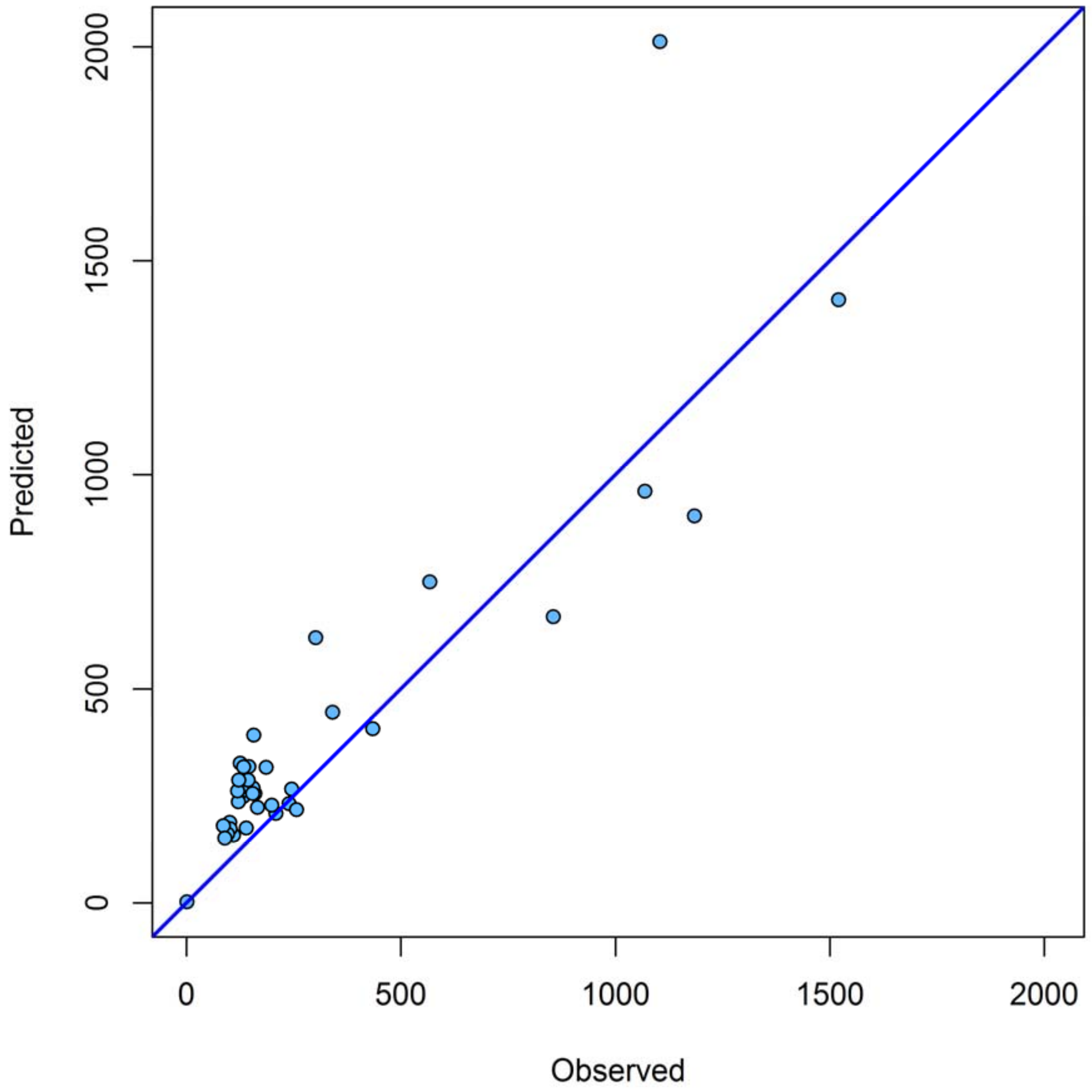

Figure A-3: Scatter Plots of the total area (×1,000 acres) invaded by water hyacinth (fall acreage) and predicted acreage using the calibrated spatio-temporal model.

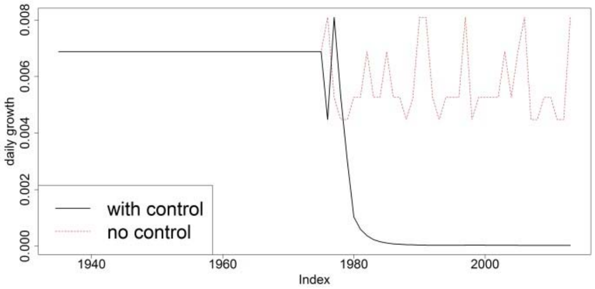

Figure A-4: Intrinsic growth rates used in the logistic growth model in Louisiana

Note: The growth rates estimated from the survey data are shown as a solid black line (1975-2013). Prior to 1975, the model uses a constant growth rate for the initiation period. The growth rates for the no control scenario are shown as a dashed red line and are resampled from the estimates between 1975 and 1978, which represent growth rates prior to biocontrol establishment.

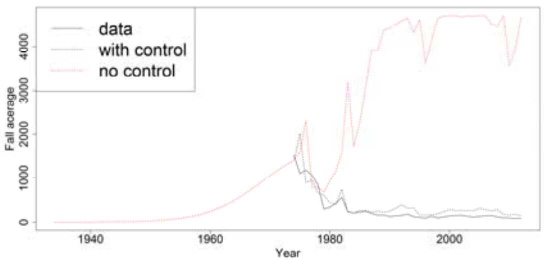

Figure A-5: Evolution of the total area (×1,000 acres) invaded by water hyacinth (fall acreage)

Note: The solid line represents an estimate of the fall invaded area for the control scenario including both herbicide and biocontrol. The black dashed line represents the predicted coverage that would have occurred with biocontrol only. The red dashed line shows the no-control scenario.

Table A-1: Simulated extent and percent cover of water hyacinth in 1987 and 2010 with and without control

	Total area covered (×1,000 acres)				Percentage of total water surface covered by water hyacinth			
	Spring 1987	Fall 1987	Spring 2010	Fall 2010	Spring 1987	Fall 1987	Spring 2010	Fall 2010
**Control**	128	266	74	148	2.1%	4.3%	1.2%	2.4%
**No-Control**	1,340	3,101	3,521	4,682	21.7%	50.2%	57.0%	75.8%

Table A-2: Parameters used in the spatio-temporal spread-model adapted to the Louisiana data

Parameter	Interpretation	Estimated	Default Value
r_y	Time varying intrinsic growth for year *y*	Yes	N/A
K_i	Carrying capacity	No	$K \times E_i / \sum E_i$ where K=6,177 thousand acres.
lags	Gap between initial occurrence and first report	Yes	From 40-70 years.
m_l	Over winter mortality during gap period	Yes	~70%, average observed between dormant seasons, 1975-1978.
m_y	Over winter mortality between 1975 and 2013	No	ratio of next spring and predicted fall acreage.
u	Kernel width	No	Twice the median distance of 50th nearest neighbor to each cell.
p	Kernel degree of freedom	No	999
λ	Smoothness of r_y	Yes	$e^{-5.3}$ to $e^{5.3}$
